# Astrocyte Involvement in Blood–Brain Barrier Function: A Critical Update Highlighting Novel, Complex, Neurovascular Interactions

**DOI:** 10.3390/ijms242417146

**Published:** 2023-12-05

**Authors:** Doina Ramona Manu, Mark Slevin, Laura Barcutean, Timea Forro, Tudor Boghitoiu, Rodica Balasa

**Affiliations:** 1 Centre for Advanced Medical and Pharmaceutical Research, “George Emil Palade” University of Medicine, Pharmacy, Science and Technology, 540142 Targu Mures, Romania; doinaramonamanu@gmail.com (D.R.M.); m.a.slevin@mmu.ac.uk (M.S.); 2 Department of Life Sciences, Manchester Metropolitan University, Manchester M15 6BH, UK; 3 Neurology 1 Clinic, County Emergency Clinical Hospital, 540136 Targu Mures, Romania; rodica.balasa@umfst.ro; 4 Department of Neurology, “George Emil Palade” University of Medicine, Pharmacy, Science and Technology, 540142 Targu Mures, Romania; 5 Doctoral School, “George Emil Palade” University of Medicine, Pharmacy, Science and Technology, 540142 Targu Mures, Romania; forro.btimea@gmail.com; 6 Psychiatry II Clinic, County Clinical Hospital, 540072 Targu Mures, Romania; tudorboghitoiu@gmail.com

**Keywords:** astrocyte, blood–brain barrier, endothelial cell, neurovascular unit, central nervous system

## Abstract

Neurological disorders have been linked to a defective blood–brain barrier (BBB), with dysfunctions triggered by stage-specific disease mechanisms, some of these being generated through interactions in the neurovascular unit (NVU). Advanced knowledge of molecular and signaling mechanisms in the NVU and the emergence of improved experimental models allow BBB permeability prediction and the development of new brain-targeted therapies. As NVU constituents, astrocytes are the most numerous glial cells, characterized by a heterogeneity that occurs as a result of developmental and context-based gene expression profiles and the differential expression of non-coding ribonucleic acids (RNAs). Due to their heterogeneity and dynamic responses to different signals, astrocytes may have a beneficial or detrimental role in the BBB’s barrier function, with deep effects on the pathophysiology of (and on the progression of) central nervous system diseases. The implication of astrocytic-derived extracellular vesicles in pathological mechanisms, due to their ability to pass the BBB, must also be considered. The molecular mechanisms of astrocytes’ interaction with endothelial cells at the BBB level are considered promising therapeutic targets in different neurological conditions. Nevertheless, a personalized and well-founded approach must be addressed, due to the temporal and spatial heterogeneity of reactive astrogliosis states during disease.

## 1. Introduction

The blood–brain barrier BBB is a complex, dynamic, selectively permeable structure that lines blood vessels in the brain. It represents the physical and metabolic barrier between the bloodstream and neuroglia from the central nervous system (CNS) parenchyma. The BBB blocks foreign substances in the bloodstream from entering brain tissue and also prevents therapeutic molecules from reaching the brain. Aspects related to BBB disruption should be known in predictions of which drug is best suited to an individual patient [1]. 

The endothelial cells (ECs) anchored to the basal lamina are critical regulators of the vascular basement membrane’s (BM) permeability through tight junctions (TJs) and adherens junctions (AJs) between adjacent ECs, which limit paracellular transport of water-soluble or polar compounds, restrict the transport of small ions, and maintain ECs’ polarity [2], whilst a collagen-rich parenchymal extracellular matrix (ECM) provides the scaffold. Pericytes modulate the contractility of the microvasculature and ultimately the BBB, and astrocytes are the ‘sensing’ cells that react to the metabolic requirements of neurons. The BM proteins, primarily secreted by local astrocytes, together with the astrocytic end-feet, almost completely surround the brain vasculature, forming glia limitans vascularis and, in this way, being responsible for maintaining the BBB’s integrity and CNS’s homeostasis [3,4]. 

In this review, we describe the complex interplay associated with maintenance of BBB integrity, which is driven by multiple factors released from heterogeneous astrocyte subtypes that consequently trigger EC activation and participation.

## 2. BBB Permeability-Regulating Proteins

Neurological disorders such as ischemia, brain trauma, and neurodegenerative diseases lead to BBB disruption, resulting in brain edema and parenchymal inflammation. In the case of injury, astrocyte phenotype shifts to a complex and heterogeneous reactive form, which can exert a protective role promoting BBB barrier recovery or, conversely, can induce EC apoptosis and decreased expression of proteins involved in BBB barrier integrity and selective permeability.

**Figure 1:**
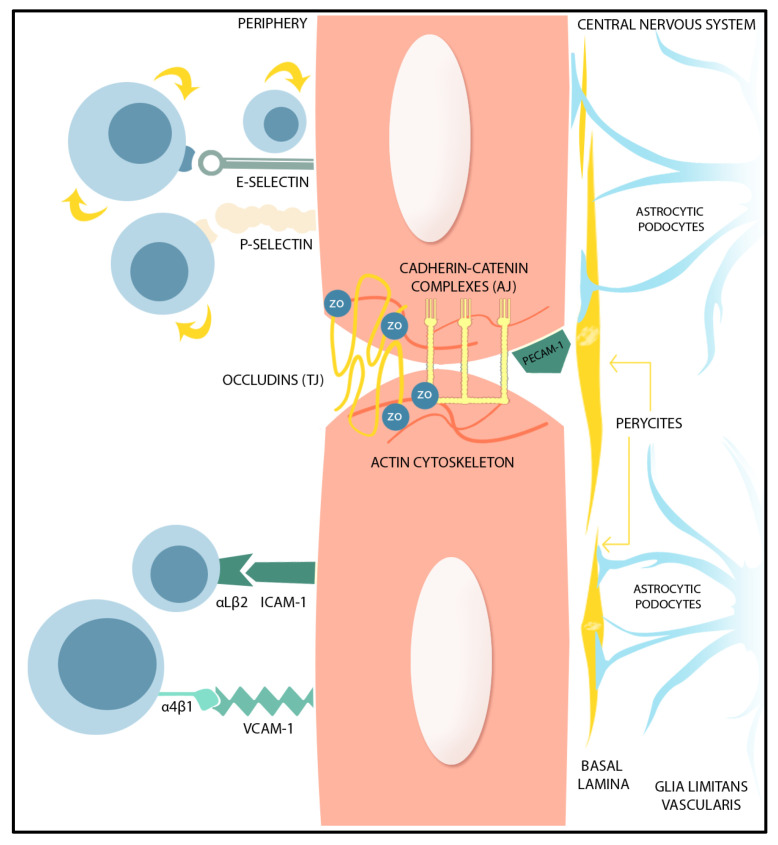
Graphical representation of the BBB structure. BBB function is supported by the presence of TJs and AJs, together with cytoplasmatic proteins, ZO. The ECs are anchored to the basal lamina. The pericytes act as contractile components in the brain’s microvascular BBB, sensible to astrocyte inputs. The parenchymal BM made from ECM secreted by astrocytes and the astrocytic end-feet form the glia limitans vascularis. (TJ: Tight Junctions, AJ: Adherent Junctions, ZO: Zonula Occludens, EC: Endothelial Cells, BBB: blood–brain barrier, BM: basement membrane, ECM: extracellular matrix).

A complex group of transmembrane proteins modulate the BBB permeability through the TJs. These include claudin, occludin, tricellulin, and junction adhesion molecules, together with cytoplasmic accessory proteins including Zonula occludens (ZO)-1, -2, -3, and cingulin. Claudin-5, claudin-3 and claudin-12 function is to seal the BBB [5,6,7], whilst oligomeric phosphorylated occludin structures decrease BBB permeability for small-molecular-weight compounds [8]. The principal components of the BBB structure are represented in Figure 1. Conversely, BBB permeability is increased after matrix metalloprotein (MMP)-mediated occludin ubiquitination or after occludin cleavage [9]. Junction adhesion molecules are involved in TJ interaction with adjacent ECs, as well as in leucocyte adhesion and migration [10], whilst the ZO-1 and -2 proteins are critical for TJ assembly and TJ anchorage to the actin cytoskeleton [11].

AJs are composed of cadherin–catenin complexes, together with TJ assembly proteins, that interact by associated adhesive cell–cell interactions. Vascular endothelial (VE)-cadherin, expressed by ECs and situated toward the basolateral side of ECs, has an essential role in this BBB permeability regulation [12].

Annexins are Ca2+ and phospholipid-binding proteins that regulate intracellular Ca2+ concentrations and traffic, as well as cellular membrane organization. Annexin A 1, expressed in pericytes and ECs, facilitates TJ function through its connection between the cytoskeleton and the plasma membrane [13]. Annexin A2, in contrast, regulates EC phenotype and AJ integrity through interaction with VE-cadherin [14].

Another class of BBB permeability-regulating proteins are the integrins, which are cell–cell or cell–ECM adhesion proteins, existing as heterodimers and formed by combination of 18 different α and β subunits. There are 24 integrin proteins with complex cellular functions, which can activate many intracellular signaling pathways [15]. They interact importantly with astrocytic end-feet. For example, the α6vβ8 integrin, expressed by astrocytes induces Transforming Growth Factor-β (TGF-β) production, which stabilizes the endothelium and astrocytic end-feet attachment within the basal lamina, in co-ordination with ECs [16]. 

Both endothelial and parenchymal BMs contain collagen IV, fibronectin, and laminins. Laminins 1 and 2 are mainly secreted by astrocytes in the parenchymal basement, and laminins 8 and 10 are secreted in the vascular BM. Astrocytic laminins regulate pericyte differentiation. Loss of astrocytic laminin decreases aquaporin-4 expression in astrocytic end-feet and down-regulates TJ protein expression [14]. Collagen IV and fibronectin are secreted by ECs, astrocytes, and pericytes, and contribute to BM stability [17]. Agrin maintains BBB integrity and function through TJ and AJ protein expression [18].

The BBB’s selective permeability relies on the fine-tuning and networking of BMECs, pericytes, and astrocytes, and dysregulation in each individual cell type may affect the function of the whole multi-cellular structure, leading to pathological events.

## 3. Endothelial Cells Interplay with Astrocytes within the Neurovascular Unit (NVU)

EC morphology, gene expression, proliferation, and transport mechanisms responsible for BBB integrity can be modified by biomechanical and biochemical signaling from the blood compartment, as well as by paracrine signaling pathways between microvascular ECs and other cells from the NVU. 

Neurovascular coupling modulates the rate of circulating blood flow to adequately match the metabolic needs of active neurons. It is regulated by a signaling network from the NVU that comprises neurons, astrocytes, and vascular cells (ECs, smooth muscle cells, and/or pericytes). 

Astrocytes are considered intracranial baroreceptors, which detect brain hypoperfusion, triggering astrocytic Ca2+ signaling and vasoactive molecule release. Adenosine triphosphate (ATP), or neurotransmitters released from active neurons, activates astrocytic P2X receptors which trigger Ca^2+^ signals, consequently inducing phospholipase D2- and diacylglycerol lipase-mediated production of arachidonic acid and the generation of prostaglandin E2 by cyclooxygenase-1. Prostaglandin E2, secreted from astrocytic end-feet, induces relaxation of pericytes by binding to the EP4 receptors [19,20]. Another mechanism involves Ca2+-activated K+ channels, which ‘switch on’ endothelial-dependent hyperpolarization, followed by vasodilation. The endothelial cells produce vasodilatation factors such as epoxyeicosatrienoic acids (by cytochrome P450 epoxygenases) [19], prostaglandin I2, and nitric oxide (NO) [21,22].

As NVU constituents, astrocytes secrete numerous paracrine factors, acting mainly on ECs, by changing the expression and distribution of TJ and AJ proteins, and ultimately affecting the BBB’s barrier properties. 

### 3.1. Astrocytic Factors Supporting BBB Integrity

The astrocyte-derived paracrine factors include factors that promote BBB integrity, such as Sonic Hedgehog (SHh), Angiopoetin-1 (ANG-1), retinoic acid (RA), Wnt Growth Factors, Insulin-Like Growth Factor (IGF-1), Glial-derived Neurotrophic Factor (GDNF), Fibroblast Growth Factor (FGF), and Apolipoprotein E (ApoE). 

Astrocytes secrete SHh [23], and BBB ECs express high levels of the Hh receptor Patched-1, the signal transducer Smoothened (Smo), as well as transcription factors of the Gli family. SHh binds to the transmembrane receptor PTCH-1, suppressing the inhibition of Smo, Gli transcription factors are transported to the nucleus, and this sequence of events promotes transcription of SHh target genes. The activation of the Hh pathway induces expression of junctional proteins and promotes BBB phenotype integrity. SHh signaling triggers changes in transendothelial electrical resistance and paracellular permeability due to increased expression of PTCH-1, claudin-3, -5, occludin, junction adhesion molecule-A, VE-cadherin, p120, and laminin [24]. 

The SHh signaling pathway is active in different brain regions, with different intensities across regions. This heterogeneity correlates with the molecular heterogeneity and differential transcriptional regulation of distinct astrocyte subsets from different brain regions. Reactive astrocytes show stimuli-dependent decreases of SHh activity, which is well defined, both spatially and temporally [25]. The astrocytes expressing Gli1 are a particular class of astrocytes, with a dynamic response to environmental stimuli. Different classes of astrocytes can be selectively activated by SHh signals originating from astrocytic or neuronal sources [26]. 

IL(interleukin)-1β produced by activated microglia suppresses SHh release from astrocytes, leading to an increased BBB permeability [23]. IL-6 and Tumor Necrosis Factor (TNF)-α trigger a decrease of TJ protein expression and an increase in reactive oxygen species (ROS).

Activation of SHh signaling decreases the level of microglial TNF-α, IL-6, and IL-1β transcripts, and increases expression of tight junction proteins ZO-1 and occludin [27,28]. *smo* gene deletion in ECs decreases expression of occludin, claudin-3, claudin-5, and ZO-1, indicating a role for SHh in EC modulation of BBB integrity [24,28]. SHh signaling exerts anti-apoptotic effects in ECs [29], whilst SHh signaling reduces intercellular adhesion molecule (ICAM)-1 expression in ECs, therefore limiting the adhesion and transmigration of immune cells [30].

High mobility group box 1 protein (HMGB1) promotes SHh release in astrocytes through the receptor for advanced glycation end products (RAGE), via downstream phosphorylation of p38, Janus kinase (JAK), and Signal transducer and the activator of transcription 3 (STAT3) [31]. 

Another member of the Hedgehog family of signaling molecules, Desert Hedgehog (DHh), regulates astrocyte association with blood vessels through PTCH binding on ECs and SMO-dependent Hedgehog target gene transcription. SHh signaling stimulates proliferation of local oligodendrocyte progenitors, adult neural stem cells, and astrocytes after injury, producing a glial scar [32].

ANG-1 is a glycoprotein produced in both ECs and astrocytes, promoting a decrease in endothelial permeability through junctional protein expression [33]. ANG-1 binds to Tie-2, a tyrosine kinase receptor expressed in ECs, leading to activation of Phosphoinositide3(PI3)-kinase/protein kinase B (AKT), Ras, and mitogen-activated protein (MAP) kinase, involved in ECs’ survival and BBB protection against damage [34,35]. ANG-1 exerts endothelial anti-apoptotic effects through activated PI3-kinase, which results in phosphorylation and inhibition of the forkhead transcription factor, or in activation of GTP-ases RhoA and Rac1. 

After ANG-1 binding, the adaptor protein Src homology-2-domain protein tyrosine phosphatase-2 is also recruited to Tie2, with downstream stimulation of Erk1/2 via Grb2, where it suppresses Vascular Endothelial Growth Factor (VEGF)-induced expression of ICAM-1 and VCAM (Vascular cell adhesion molecule)-1. ANG-1 counteracts VEGF-induced endothelial permeability by inhibiting Src-mediated phosphorylation of VE-cadherin [36,37]. Xia et al. proposed that ANG-1 up-regulates ZO-1 and occludin after permanent ischemic damage [38]. 

ANG-1/Tie-2 signaling maintain TJ integrity through PTPN-2 (protein tyrosine phosphatase non-receptor type 2), which regulates tyrosine dephosphorylation of occludin in response to ANG-1. ANG-1 prevents thrombin-mediated tyrosine phosphorylation of occludin in a PTPN-2-dependent manner, favoring occludin and ZO-1 interaction, and TJ integrity recovery [39]. 

When BBB-disrupting factors, such as VEGF, are co-expressed with ANG-1, the barrier integrity is enhanced [40].

Conversely, astrocyte-derived ANG-2 participates in the early phases of disease–BBB disruption, inducing vessels tightening through TJ formation. Astrocytes also produce the angiotensin-converting enzyme-1 (ACE-1) that converts ANG-1 into ANG-2. ACE-1 acts on type 1 angiotensin receptors expressed by BMECs. In the CNS, activation of type 1 angiotensin receptor restricts BBB permeability and stabilizes junctional protein function by promoting their recruitment into lipid rafts [41,42]. 

RA, a metabolite of vitamin A, is synthesized from retinol by retinaldehyde dehydrogenase, and is highly expressed in reactive astrocytes, triggering enhanced astrocytic RA synthesis [43]. RA increases expression of ZO-1 and VE-cadherin [44], and decreases VCAM-1 expression in brain microvascular ECs during inflammation [45]. 

In a human in vitro BBB model comprising brain microvascular endothelial cells (BMECs), pericytes, astrocytes, and neurons, RA-treated BMECs expressed VE-cadherin, occludin, and claudin-5 [46,47]. 

RA reduces β-catenin expression through transcriptional suppression. Moreover, RA promotes an increase in the phosphorylation of β-catenin (Ser33/Ser37/Thr41) through RA receptor and protein kinase C (PKC) activity, followed by proteasome-mediated degradation of phosphorylated β-catenin. However, Bonney et al. showed that RA inhibition of endothelial Wnt signaling has any effect on claudin-5 expression, due to ectopic vascular Wnt-β-catenin signaling [48]. 

The Wnt/β-catenin pathway and Wnt growth factors released by astrocytes influence BBB integrity, as shown by Guérit et al. hGFAP-Cre-mediated deletion of the evenness interrupted (Evi) gene, a mediator of Wnt growth factor secretion, leads to brain edema and tracer extravasation. However, effects on EC junction protein expression or transendothelial electrical resistance measurements were not observed. It was presumed that β-catenin-mediated Wnt signaling in ECs exceeded Wnt secretion from astrocytes [49]. 

Canonical and non-canonical Wnts can exert opposing effect in cells from the NVU. The non-canonical Wnt pathways or non-β-catenin-mediated Wnt pathways are involved in cell polarization, cell survival, inflammatory response, and cell migration. The canonical or β-catenin-mediated Wnt signaling pathway is activated after Wnt growth factor binds to Frizzled (Fzd) and the low-density lipoprotein receptor-related protein 5 or 6 (LRP5/6). Wnt binding to its receptor results in the recruitment of the disheveled (Dsh) protein and β-catenin phosphorylation complex, formed by glycogen synthase kinase 3β (GSK-3β), Axin, adenomatous polyposis coli, and casein kinase-1. After the inhibition of β-catenin phosphorylation complex, cytoplasmic β-catenin is translocated to the nucleus, resulting in the transcription of target genes involved in angiogenesis, vascular integrity, and BBB TJ expression. Increased β-catenin activation is followed by endothelial expression of claudin-3 and claudin-5 and reduced BBB permeability. In the absence of Wnt growth factors, β-catenin is phosphorylated by the protein complex, resulting in β-catenin proteasome-dependent degradation [50].

Ligands inducing Wnt/β-catenin signaling suppression increased both caveolin-1-mediated transcellular transport in ECs and BBB transcellular permeability. Wnt factors secreted by astrocytes were associated with low caveolin-1 expression and decreased transcellular vesicular traffic in brain ECs. A reduction of Wnt factor release from astrocytes decreased Wnt pathway target gene expression in ECs and astrocytes. When astrocytic Wnt factor was suppressed, end-feet coverage of brain microvessels was impaired [49]. 

IGF-1 is a neurotrophic factor produced in astrocytes, neurons, ECs, and other glia. Bake et al. reported that astrocyte-derived IGF-1 exerted protective effects against EC death, thus attenuating BBB disruption [51].

GDNF produced in astrocytes increases claudin-5, occludin, and ZO-1 expression. Therefore, GDNF exerts barrier-protective effects in the BBB via TJ protein up-regulation in EC [52,53,54]. 

Astrocytes, microglia, ECs, leukocytes, neurons, and other cells interact at different stages of the response to injury. In vivo administration of FGFs after injury can affect astrocyte phenotype directly or through changes in other cell types. FGFs act in injury in a stage-dependent manner to either activate or suppress astrocyte activation. Deletion of FGFs, β-integrin, and sHh lead to reactive astrogliosis [55]. FGF receptors (FGFRs) are expressed in neurons, astrocytes, and oligodendrocytes. FGF ligand binding on FGFRs activates downstream PLC (Phospholipase) γ, MAP kinase/extracellular signal-regulated kinase (ERK), and STAT3 pathways. FGF-2 and FGFRs are involved in communication between degenerating neurons, microglia, and astrocytes. FGF signaling provides a supportive ECM and increased astrocytic proliferation [56]. Cyclin D1 and E are required for cell cycle progression and proliferation. c-Myc regulates gene expression through binding on the enhancer sequence and recruiting histone acetyltransferases. FGF-2 increases Cyclin D1 and c-Myc expression and mediates astrocyte proliferation [57]. Kang et al. showed that FGF8 biding on FGFR3 was responsible for an astrocyte morphology with more branches than that produced via FGF2 signaling. FGFR3 reduces astrocyte branch formation and minimizes hypertrophic responses in the site of reactive gliosis [58]. A study performed by Reuss et al. with mice lacking FGF-2 or FGF-5 showed that FGF signaling regulates astrocytes differentiation in a region-specific manner, this being demonstrated by reduced levels of glial fibrillar acidic protein (GFAP) in FGF-2- and FGF-5-deficient mice. Reduced levels of GFAP were also observed in perivascular astroglial end-feet, accompanied by albumin extravasation from brain capillaries and reduced levels of the TJ proteins ZO-1 and occludin in ECs from brain capillaries. These results showed that FGF signaling may have a role in the regulation of BBB permeability in vivo [59,60,61]. 

Astrocytes represent a major source of apolipoprotein E (ApoE) in the brain. APOE4 disrupts the integrity of the BBB by activating the cyclophilin A (CypA)NF-κB-matrix metalloproteinase 9 pathway in pericytes. APOE3, produced by astrocytes, suppresses APOE4 signaling and counteracts APOE4-associated BBB breakdown [62,63,64].

### 3.2. Astrocyte-Derived Factors Increasing BBB Permeability

Astrocyte-derived factors capable of promoting BBB permeability in neurological disorders are VEGF, NO, matrix metalloproteinases (MMPs), Glutamate, and Endothelins (ETs). 

VEGF/VEGF receptor (VEGFR)2 signaling triggers vascular leakage through downstream pathway PI3K/AKT-endothelial nitric oxide synthase (eNOS) in ECs. The PI3K/AKT pathway phosphorylates eNOS and activates eNOS enzymatic functions leading to NO production. Oxidative stress mediates ROS production, and ROS reacts with NO to synthesize peroxynitrite. ROS signaling inhibits PHD2 activity, leading to Hypoxia-inducible factor (HIF)-1α and nuclear factor kappa light chain enhancer of activated B cells (NF-kB) transcription factor stabilization and activation, consequently up-regulating the expression of downstream related genes and proteins [NF-kB (IL-1β, TNF-α, IL-6, ICAM-1, and VCAM-1)] and HIF-1α-mediated VEGF expression. ONOO-provokes detachment of VE-cadherin from ECs, whilst the VEGFR2-mediated Src–Rac1–p21-activated kinase (PAK) pathway phosphorylates VE-cadherin, resulting in dissociation of AJs between ECs. HIF1α- and NF-κB-mediated gene expression facilitates BBB breakdown, inflammatory responses, and glial scarring [65]. VEGF down-regulated endothelial expression of both claudin-5 and occludin is implicated in lowering of BBB integrity. VEGF down-regulation of claudin-5 and occludin occurs via VEGFR2 signaling through PLCγ and its effector, eNOS. STAT3 is also a potential modulator of HIF-1α-mediated VEGF expression [66]. 

However, Baumann et al. found that astrocyte-derived HIF-1α-dependent paracrine signaling did not contribute to the modulation of EC barrier function under normoxic or hypoxic conditions. Astrocytes prevented hypoxia-induced increased permeability, but through an HIF-1 independent pathway, which triggers increased astrocytic VEGF levels [67]. The effects of astrocytic VEGF on ECs has been described previously as being partially attributed to NO generation in these cells, through a NO synthase/cGMP-dependent pathway [68,69,70]. 

Glutamate is a major excitatory transmitter which exerts its excitatory effects via glutamatergic receptors, including the N-methyl-D-aspartate (NMDA) receptor and the α-amino-3-hydroxy-5-methyl-4-isoxazolepropionic acid (AMPA) receptor. Astrocyte-derived glutamate binds on NMDA receptors from ECs and induces vasodilatation that is dependent on NOS-3. NMDA receptors are ionotropic receptors and act as ion channels after glutamate binding, increasing the intracellular calcium concentration. Elevated cytoplasmic Ca^2+^ triggers mitochondrial production of ROS, disrupting endothelial junctions and the cerebral endothelial barrier. ROS can mobilize intracellular Ca^2+^ stores and lead to the opening of calcium channels, both of which contribute to endothelial barrier leakage [71].

MMPs are zinc-endopeptidases released from reactive astrocytes, and are involved in degradation of endothelial TJ and ECM remodeling. The pathological role of MMPs in acute and chronic neurodegenerative disorders is reflected by BBB disruption [72,73]. Claudin-5 and occludin degradation occurs through MMP-2 action in the early phase of BBB disruption, and through MMP-9 in the delayed phase [74]. Increased MMP-9 expression is responsible for EC apoptosis through laminin degradation following subarachnoid hemorrhage in rats [75]. MMP-9 and MMP-2 contribute to caspase3-mediated brain EC death after hypoxia-reoxygenation [76]. 

Glutamate released during epileptic seizures triggers increased MMP-2 and MMP-9 expression and activity, leading to decreased TJ protein levels and thereby resulting in BBB leakage [77,78].

ET-1, -2 and -3 are endogenous vasoconstrictors that regulate EC function through Endothelin receptor (ETR) type A and type B. ETRB is expressed in astrocytes and up-regulated in brain disorders. ET-1 is synthesized by ECs, macrophages, cardiomyocytes, astrocytes, microglia, and neuronal cells [79]. 

ET-1 activates the ETRB receptor, followed by PLC and then PKC activation. Activation of the PKC pathway leads to ROS release, which increases the production of MMP-9. Exposure of astrocytes to ET-1 also triggers the up-regulation of NO synthase and NO production [80]. Increased production of MMP-9 and VEGF stimulated by ETs induces brain edema [81,82,83,84].

**Figure 2:**
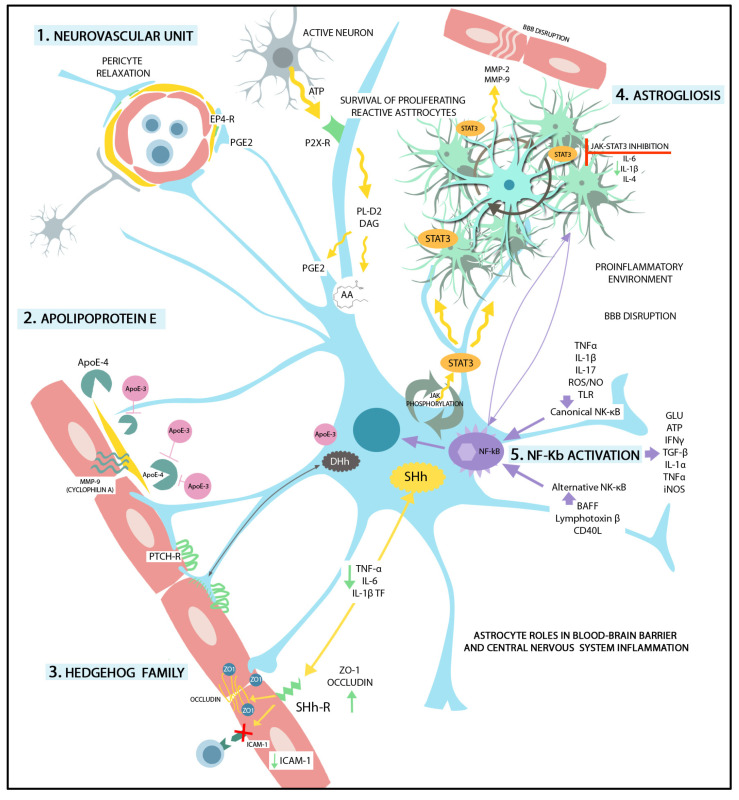
Graphical representation of the most important astrocytic roles in BBB and CNS function. (1) Neurovascular unit. ATP from active neurons activates astrocytic P2X receptors that induce PL-D2 and diacylglycerol lipase-mediated production of arahidonic acid and generation of prostaglandin E2. Prostaglandin E2 is secreted by the astrocytic end-feed and induces pericyte relaxation by binding to EP4-R. (2) Apolipoprotein E (ApoE). The astrocytes are the main source of ApoE in the CNS. ApoE-4 activates cyclophilin A (MMP-9) in the pericytes which disrupts the BBB. ApoE3, produced by astrocytes, blocks the APOE-4-associated BBB disruption. (3) Hedgehog Family. Sonic Hedgehog (SHh). SHh signaling through endothelial receptors decreases TNF-alfa, IL-6, and IL-1b transcription factors, increases the expression of TJs (occludin, ZO-1, etc.), and reduces ICAM-1 expression on ECs. Desert Hedgehog (DHh) regulates the binding between astrocytic end-feet and protein-patched receptors (PTCH-R). (4) Astrogliosis. STAT3 activation in reactive astrocytes (via phosphorylation by Janus kinase) leads to reactive astrogliosis. STAT3 regulates the survival of proliferating reactive astrocytes. Additionally, MMP-2 and -9 are released from reactive astrocytes, favoring BBB disruption. Janus kinase-STAT3 inhibition has been shown to reduce levels of IL-6, IL-1b, and IL-4. (5) NF-Kb activation. Astrogliosis is accompanied by NF-kB activation in reactive astrocytes which triggers BBB disruption. The canonical NK-κB pathway is induced by TLRs, pro-inflammatory cytokines, ROS, and NO. The alternative NK-κB pathway is activated by the B cell activating factor of the TNF family (BAFF), lymphotoxin β, and CD40L. NF-kB pathway activation leads to glutamine, ATP, IFNγ, TGF-β, IL-1α, TNFα, and iNOS production, promoting a pro-inflammatory environment and leading to BBB disruption.

In summary, astrocytes, as the main secretory cells in CNS, produce paracrine factors that can be expressed at low levels or even not expressed in a healthy brain. Under pathological conditions, the expression of BBB permeability-inducing factors is increased in the reactive astrocytes, as summarized by Figure 2. After CNS injury, the astrocytes’ functional polarization may also be set to up-regulate BBB integrity-restoring factors. Local environmental stimulation may lead to changes in the secretion of paracrine factors by astrocytes, triggering a dynamic regulation of the BBB. Astrocytic soluble factors with physiological roles in BBB maintenance, as well as soluble factors secreted in reactive astrocytosis triggering BBB disruption and vascular inflammation, are described in Table 1. Several potential molecular mechanisms underlying BBB structural and functional modulation in health and disease under astrocyte control are also summarized. 

## 4. Reactive Astrogliosis in BBB Dysfunction

Oxidative or chemical stress and a pro-inflammatory milieu elicit astrocyte reactivity, reflected in transcriptional changes. Astrocyte exposure to a multitude of extracellular signals induces the hypertrophy, proliferation, and secretion of pro-inflammatory molecules. The intensity and duration of transcriptional changes depend on the type and power of different stimuli eliciting an array of changes, from some reversible ones to glial scar formation. Astrocytes exhibit dynamic differences in morphology, physiological properties, function, and response to injury and disease [91]. 

Escartin et al. have defined the term “reactive astrogliosis’’ which describes changes in transcriptional regulation, as well as biochemical, morphological, metabolic, and physiological states in response to pathological stimuli. In contrast, astrocyte plasticity has been defined as changes in astrocyte gene expression and phenotype in response to physiological signals [92]. 

Reactive astrocytes have pro-inflammatory and oxidative profiles, and increased expression of vasoactive mediators, triggering endothelial junction disruption, immune cell infiltration, and Endothelial β-catenin down-regulation of WNT/β-catenin signaling, leads to decreased levels of TJs proteins and disruption of intercellular junctions, enhancing BBB destabilization and breakdown [62,93].

Activation of NF-κB in astrocytes by deletion of IκBα triggers brain inflammation and BBB disruption. NF-κB inhibition in astrocytes, conferred by transgenic expression of a degradation-resistant form of IκBα (IκBα-dn), elicits down-regulation of chemokine C-C motiv ligand (CCL)2, CCL5, ICAM-1, VCAM-1, and Itg that are essential for leukocyte adhesion to brain microvessels and migration into the CNS through BBB [85,90]. 

Ceramide-derived sphingolipids trigger NF-kB pathway activation, and reactive astrocytes up-regulate expression of the sphingolipid receptor S1PR1. Lactosylceramide (LacCer) synthesis is enhanced in reactive astrocytes due to NF-kB-dependent increased expression of β-1,4-galactosyltransferase 6 (B4GALT6). In turn, LacCer enhances interferon (IFN) regulatory factor 1 (IRF-1) and NF-kB recruitment to the promoter regions of *ccl2*, *csf2*, and *nos2* involved in pro-inflammatory states and BBB disruption [94].

NF-kB pathway activation is followed by glutamate and ATP release and gene transcription of prostaglandin D2, IFNγ, TGF-β, IL-1α, C1q, and TNFα, and iNOS. 

Ligand-activated transcription factor aryl hydrocarbon receptor (AHR) limits NF-kB signaling after activation by small molecules originated from cellular and commensal flora metabolisms. In turn, AHR inactivation in astrocytes increases expression of pro-inflammatory cytokines (IL-6, IL-12, IL-23, GM-CSF), CCL2, CCL20, and CXCL10, NO, and molecules associated with astrocyte reactivity, Vimentin and GFAP [95,96]. 

NF-κB activation in astrogliosis decreases expression of TJs, AJs, and endothelial efflux transporters, but meanwhile increases endothelial inflammatory response and oxidative stress [86]. In acute inflammatory reactions, NF-κB is also activated in ECs through IL-1β and TNF-α, leading to decreased expression and an altered cellular localization of TJ proteins ZO-1 and claudin-5. Inhibition of IKK or protein kinase C zeta (PKCζ) blocks this effect on TJ proteins [97]. 

Conversely, as Ridder et al. described, NF-κB basal activity is needed for brain EC survival and function. Inflammatory mediators activate the protein kinase TAK1 and subsequently TAK1 stimulates IKK. TAK1 and the NEMO component of IKK can prevent EC death independent of the NF-Κb pathway components p65 and IKK2. This NF-κB-mediated endothelial pathway seems to revoke the increased permeability of the BBB in the presence of inflammation [98].

STAT3 may also switch gene transcription in reactive astrocyte subsets in a context-dependent manner [99]. STAT3 activation in astrocytes via phosphorylation by JAK is associated with reactive astrogliosis. STAT3 regulates the production of cytokines and chemokines in reactive astrocyte. Inhibition of the JAK–STAT3 pathway reduces mRNA levels of IL 6, IL-1b, IL-4, and VEGF in cell cultures [100]. Lipopolysaccharide (LPS)-mediated induction of the chemokines CCL20, CX3CL1, CXCL5, and CXCL10 has been described in astrocytes [101]. TNF induces a reactive astrocyte phenotype that result in BBB dysfunction through activation of STAT3 and increased expression of *serpinA3*, which encodes alpha 1-antichymotrypsin [102].

Disease-specific ligands stimulate different receptors with different intensities, durations, and frequencies, activating several JAKs, which results in different transcriptional and functional profiles. In addition, STAT3 can form heterodimers with other STATs [103]. After STAT3 signaling pathway up-regulation, reactive astrocytes may change their response profiles, resulting in the secretion of anti-inflammatory cytokines and neurotrophins. Astrocytes secrete thrombospondins (THBS1 and THBS2), aquaporin-4, HMGB1, and β2 integrin, switching to a neuroprotective function. Astrocytes may also express trophic factors such as brain-derived neurotrophic factor (BDNF), VEGF and FGF-2. In contrast, antigen presentation H2-D1, guanylate-binding protein 2 induced by IFNγ/TNF/IL-1 (Gbp2), and immunophilin FK506-binding protein 5 are down-regulated. As already mentioned, activation of the FGF-2/fibroblast growth factor receptor1 (FGFR1) pathway inhibited astrocyte-mediated neuroinflammation and BBB disruption, and reduced astrocyte neurotoxicity both in vitro and in vivo [104,105,106].

Calcineurin (also known as protein phosphatase 3), a serine/threonine-protein activated by increased intracellular Ca^2+^ levels, binds and dephosphorylates nuclear factor of activated T-cells (NFAT), resulting in NFAT nuclear translocation in the nucleus followed by conditioned activation of gene transcription. NFAT signaling relies on the activation of PLC through different receptors, like T-cell receptor (TCR) or B-cell receptor (BCR). This activation leads to the release of PI3 and diacylglycerol. The PI3 is especially important for calcium influx because it binds to a PI3 receptor located in the membrane of ER. Calcineurin-expressing reactive astrocytes are present in neurodegenerative diseases [107,108]. 

Reactive astrocytes can affect BBB endothelial integrity through secreted proteins [30,109,110]. Astrocytes have also been shown to secrete factors that interfere with leukocyte recruitment and migration into the CNS through BBB [111,112]. The presence of an increased number of reactive astrocytes, which is disease-associated, is involved in BBB disruption [113,114]. Contrarily, some studies have shown that reactive astrocytes are sustaining BBB function after neural injury [115,116].

## 5. Astrocyte Functional Identity Is Driven by Transcriptional and Epigenetic Changes

The astrocyte end-feet are an important component of BBB, together with brain endothelial cells, vascular smooth muscle cells, pericytes, and the vascular basement membrane. The dynamic changes of BBB functions are fundamentally influenced by the direct influence of the heterogeneous astrocyte subsets. A mechanistic binary division of astrocytes into neurotoxic (A1) and neuroprotective (A2) phenotypes is considered outdated. The research on reactive astrocytes’ influence on BBB permeability is actually based on multiple functional parameters used to obtain relevant data concerning the detrimental versus protective roles of the astrocytes.

Astrocyte heterogeneity and plasticity in different brain regions, as well as within a brain region, are owed to developmental and context-based gene expression profiles. The large-area spatial transcriptomic (LaST) map developed by Bayraktar et al. quantifies different gene expressions at the single-cell level. With this approach, the authors demonstrated gene expression gradients in astrocytes across large tissue areas in mouse brains. Whole tissue sections were examined with a high 3D resolution in spinning-disk confocal microscopy, then single-cell (sc)RNA-seq data, followed by spatial reconstruction analysis, confirmed the presence of superficial, mid, and deep astrocyte layers in the adult mouse cortex. Screening 46 candidate astrocytic genes with potential differential expression, the authors found layer-independent heterogeneity, detected by clustering of scRNAseq data, and layer-dependent heterogeneity, demonstrated by single-molecule fluorescent in situ hybridization (smFISH) and spatial reconstruction of scRNA-seq data [117]. 

Transcription factor motif analysis showed the presence of multiple transcriptional layers in astrocytes across different brain regions. Lozzi et al. identified generalized astrocytic transcriptional regulators, as well as region-specific transcription factors in adult astrocytes. The region-specific transcription factors control regulatory networks and contribute to the distinct molecular signatures and functional profiles of astrocyte heterogeneity [118]. 

In neurodegenerative diseases, a spectrum of reactive-astrocyte phenotypes from different brain regions respond to pathological stimuli at a given time point, and thus astrocytic involvement might also be disease stage-dependent [119]. 

sc-RNA-seq and single-nucleus (sn)-RNA-seq combined with multidimensional data and clustering analysis described distinct and complex stage-dependent transcriptomic profiles in chronic neurodegenerative disease. Further, sc-RNA-seq and sn-RNA-seq highlighted astrocytes exhibiting alternative phenotypes, regenerative or degenerative, in a context-dependent manner, across different CNS regions [120]. 

Extrinsic signals and/or interaction with CNS-resident or non-resident cells can trigger alteration of epigenetic states that, in turn, can modify intrinsic transcriptional programs, with long-term influence on astrocyte identity [121,122].

**Figure 3:**
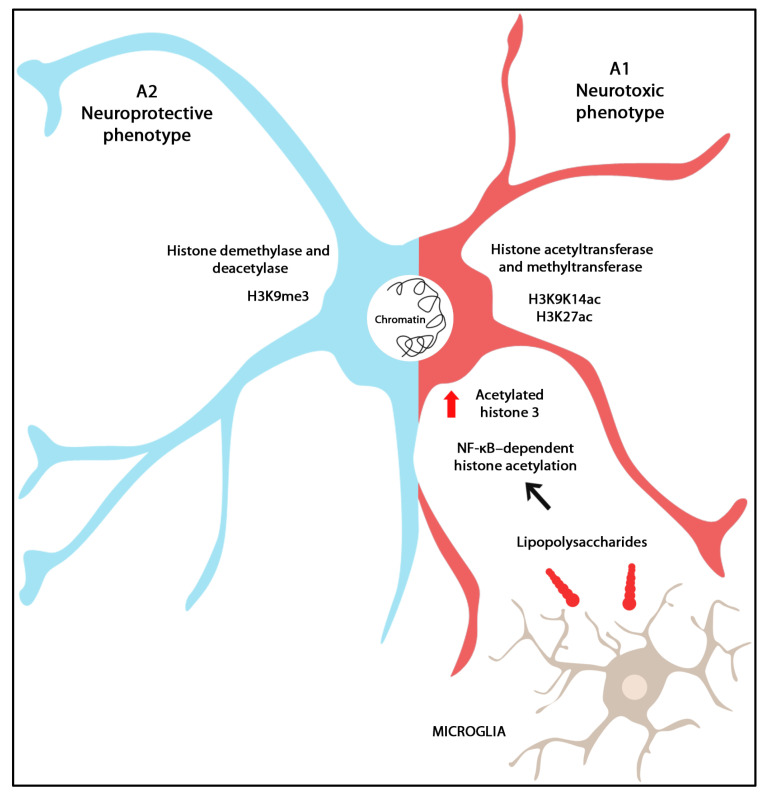
Graphical representation of the epigenetic landscape of astrocyte reactivity. Inside the CNS, the astrocytes present two phenotypes: A1, neurotoxic and A2, neuroprotective. The chromatin structure can be modified by alterations in the histone structures, catalyzed by histone acetyltransferases (HATs) and histone methyltransferases (HMTs), and diminished by histone demethylases (HDMs) and histone deacetylases (HDACs). When exposed to LPS in the presence of microglia, reactive astrocytes exhibit elevated levels of acetylated histone 3. The acetylation of histone 3, driven by NF-κB signaling, particularly at active transcriptional sites, serves as a hallmark indicating a pro-inflammatory astrocyte phenotype.

Gene transcription is a complex process which is dependent not only on chromatin structure but also on its accessibility for transcription regulators. The assay for transposase-accessible chromatin (ATAC-seq) is used for identifying genome-wide accessible regions of chromatin [123]. Genome-wide chromatin analysis highlights the importance of spatial chromatin architecture. Structures like chromatin loops can bring distant promoters and regulatory elements involved in transcriptional regulation into close proximity [124]. Chromatin structure can be modified through histone rearrangements as follows: it can be (1) catalyzed by histone acetyltransferases (HATs) and methyltransferases (HMTs) and (2) reduced by histone demethylases (HDMs) and deacetylases (HDACs). Increased levels of acetylated histone 3 at lysins 9, 14, and 27 (H3K9K14ac and H3K27ac, respectively) were detected in reactive astrocytes after LPS exposure in the presence of microglia. Additionally, NF-κB-dependent histone acetylation in active transcriptional sites is a marker for pro-inflammatory astrocyte phenotypes. H3K9me3 is a marker of transcription silencing and decreased astrocyte reactivity initially derived from LPS exposure. Moreover, some astrocyte subsets are characterized by active transcription, whereas others are repressed due to H3K27ac and H3K9me3 changes [125] (Figure 3).

Histone phosphorylation on serine, threonine, and tyrosine is involved in chromatin condensation and transcription silencing. Histone ubiquitination is associated with genome stability, cell cycle, and transcription. Other histone modifications such as crotonylation, lactylation, and serotonylation, identified by mass spectrometry, are involved in neurodevelopment and neurological disease [126].

Another epigenetic mechanism involved in gene transcription regulation is DNA methylation. Cytosine methylation represses gene expression through the addition of methyl groups within CpG dinucleotides, and 5-methylcytosine is an epigenetic marker found in CpG islands from about 60% of promoter sequences. Methylated CpG islands from gene promoters prevent interactions with transcriptional regulators or, conversely, recruit proteins involved in gene repression, such as co-repressors [127]. 

DNA methylation and open chromatin maps identified with genome-wide next generation sequencing (NGS) techniques showed functional changes of the promoters and distal or proximal cis regulatory regions in astroglia from different brain regions in young adult mice, leading to functional, epigenomic, and regional diversity. Hierarchical clustering of the total mRNA-Seq, ATAC-Seq, and RRBS datasets highlighted transcriptomic- and epigenomic-specific changes from cortical to cerebellar astrocytes [128].

There is no clear demarcation between the pro- and anti-inflammatory status of astrocyte subsets in the brain, and the differential expression of non-coding RNAs occurring in astrocytes contributes to their heterogeneous phenotypes and context-dependent response. 

Non-coding RNAs represent an epigenetic mechanism that leads to gene silencing, affecting protein translation without altering the DNA sequence. MicroRNAs (miRNAs) are a large group of small endogenous molecules of single strand non-coding RNA, approximately 21–25 nucleotides in length, and their fundamental role is to regulate gene expression at the post-transcriptional level. They act as negative regulators by binding through the 3′ untranslated region (UTR) of mRNA targets and determining its degradation and/or the inhibition of its translation. MiRNAs can either directly preserve BBB integrity by targeting endothelial junction protein translation, or indirectly by down-regulating NF-Kb, mitogen-activated protein kinases, and PI3/Akt signaling pathways to reduce inflammation and apoptosis, or by promoting the expression of ANG-1/Tie-2 axis in ECs. Additionally, miRNAs may also have an impact on the crosstalk between brain ECs and supporting cells, which is critical for the maintenance of BBB function [129].

Kong et al. showed pro-inflammatory cytokine expression changes through miRs after myeloid-related protein (MRP) 8 binding to Toll-like receptor (TLR) 4 in astrocytes. After TLR4 activation, the Myd88-IRAK4-IRAK2/1 complex is formed, followed by TNFR-associated factor 6 (TRAF6) activation and IL-1β, IL-6, and TNF-α gene transcription. miR-132 is a cyclic AMP-responsive element binding (CREB)-regulating miRNA, which targets IRAK4, further suppressing IL-1β and IL-6 release from MRP8-activated astrocytes. Astrocytic miR-146a increased significantly after MRP8 exposure and targets IRAK1, IRAK2, and TRAF6, triggering IL-1β and IL-6 expression. The authors also showed that regulation of TNF-α production is associated with miR-155 [130]. 

Astrocyte activation induces up-regulation of miR-155, which, by inhibiting the mRNA of SOCS-1, determines pro-inflammatory cytokine expression. SOCS-1 regulates the immune response through direct inhibition of JAK and consequent inhibition of STAT [131,132].

Korotkov et al. showed that miR-155 could be induced in astrocytes by pro-inflammatory TNF-α through NF-κB and activator protein 1 (AP-1) pathways, and that this effect is potentiated in the presence of miR-142. Increased expression of IL-1β and PTGS2 was seen in astrocytes following stimulation via the medium from the miR-142-overexpressing cells [133].

**Figure 4:**
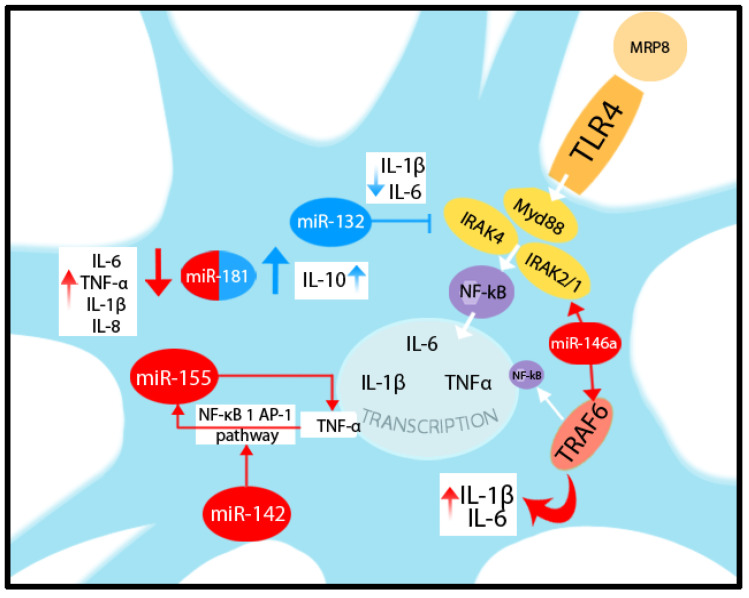
Graphical representation of some miRNAs involved in astrocytic pro and anti-inflammatory function. MRP8 binds to TLR4 in astrocytes, activating the Myd88-IRAK4-IRAK2/1 complex and TRAF6, leading to IL-1β, IL-6, and TNF-α gene transcription. miR-132 targets IRAK4, suppressing IL-1β and IL-6 release from MRP8-activated astrocytes. miR-155 is induced by TNF-α through the NF-κB 1 AP-1 pathway, augmented by the presence of miR-142; furthermore, miR-155 regulates TNF-α production. miR-146a targets IRAK1/2 and TRAF6 inducing IL-1β and IL-6 expression. Low levels of miR-181 trigger pro-inflammatory cytokine release (IL-6, TNF-α, IL-1β, and IL-8) and high levels stimulate anti-inflammatory IL-10 production from the astrocyte.

miR-181 expressed in astrocytes has an important role in the post-transcriptional regulation of reactive astrogliosis phenotypes. miR-181 directly targets the mRNA of Bcl-2, a mitochondrial membrane-associated protein that inhibits apoptosis, and heat-shock protein 70, expressed in response to cellular stress. A remarkable change in astrocyte phenotype has been observed after LPS exposure through miR-181 down-regulation. A reduced level of miR-181 triggers pro-inflammatory cytokine release, including leukemia inhibitory factor or LIF, IL-6, TNF-α, IL-1β, IL-8, and HMGB1. In contrast, levels of the anti-inflammatory cytokine IL-10 increase in astrocytes when miR-181 is over-expressed. Knockdown of miR-181 resulted in elevated FGF2 levels in LPS-treated astrocytes [134]. miRNAs involved in astrocytic pro and anti-inflammatory function are depicted in Figure 4.

Mouse brain microvascular ECs in co-culture with astrocytes were subjected to oxygen–glucose deprivation followed by reperfusion in the absence or presence of VEGF. VEGF increased the level of LOC102640519, which in turn positively regulated the expression of HOXC13 and negatively regulated TJ-associated proteins (ZO-1, Occludin, and Claudin-5), leading to BBB leakage [135].

In physiological conditions, the molecules from the cargo of astrocyte-derived exosomes contribute to the maintenance of CNS homeostasis. Astrocytic exosomes are enriched with miR-195, which improves BBB integrity [136].

Reactive astrocytes accelerate secretome release, including extracellular vesicles, which transfer neurotoxic molecules that trigger neurodegeneration, or pro-inflammatory molecules causing neuroinflammation. Astrocyte-derived extracellular vesicles pass throughout the BBB, reaching the periphery, where pro-inflammatory factors promote leukocyte migration from the periphery to brain parenchyma [137].

Jovičić et al. showed that astrocytes exclusively secrete 12 miRNAs via exosomes; meanwhile, 217 different miRNAs have been exclusively detected in astrocytes. Of these, 61 miRNAs were more than two-fold enriched in astrocytes compared to exosomes, and 42 miRNAs had higher than two-fold enrichment in exosomes compared to astrocytes. The authors also suggested the existence of a yet-undecrypted mechanism through which miRNAs are selected for inclusion or exclusion from exosomes [138].

## 6. Conclusions

BMECs are endowed with a complex network of intercellular tight and adherens junctions which, together with a low transcytosis rate and a specific transport system, confer to the BBB the role of a selectively permeable barrier. The structural and functional integrity of this barrier ensures the protection of the CNS from endogenous and exogenous threats, maintaining the brain parenchyma’s homeostasis and immune privilege, effective for neuron functioning.

BBB permeability is affected by the surrounding microenvironment, which is dynamically changed by the interactions and signaling between neurons and non-neuronal cells such as ECs, pericytes, and astrocytes in NVU.

Astrocytes form the perivascular end-feet at the BBB and secrete many factors that modulate the integrity of this barrier in the healthy adult brain, as well as factors that change permeability in pathological conditions.

Under neurological disorders, a loss in the permeability of the barrier is associated with phenotypical changes in both the ECs and astrocytes. Astrocytic structural and functional changes occur via signaling events modulated in a disease-dependent manner, leading to reactive astrocytosis. Reactive astrocytes exhibit temporal and spatial heterogeneity and a spectrum of activation states via differentially expressed genes and via changes in gene expression at the post-transcriptional level under non-coding RNA regulation. 

In this review, we showed detailed aspects of ECs, their interaction with astrocytes in NVU, and the subsequent effects on BBB permeability. We also highlighted mechanisms leading to astrocyte heterogeneity, these being more complex than the simple phenotype dichotomy of astrocytes with neurotoxic and pro-inflammatory, or neuroprotective and anti-inflammatory features. The BBB microenvironment is a complex and versatile one, in which heterogeneous astrocyte subpopulations have a central role, contributing to the known multifaceted neurological pathology and patient-personalized responses to therapy.

## Figures and Tables

**Table 1:** Astrocytic factors involved in BBB structural and functional modulation.

Factors Promoting BBB Integrity	Mechanism of Action in Brain ECs	References
Sonic Hedgehog	Activates PTCH1 and SMO followed by GLI1 nuclear translocation. Increases expression of junctional proteins claudin-3, -5, occludin, junction adhesion molecule-A, VE-cadherin, p120, and laminin expression. Decreases the level of microglial TNF-α, IL-6, and IL-1β, CCL2, and of ICAM-1 in ECs.	[23,24,27,28,30,85]
Angiopoetin-1	Binds on angiopoietin-1 receptor Tie2, activation of PI3K /AKT-myocyte enhancer factor-2 (MEF2)-Krüppel-like factor 2 (KLF2) pathway stabilizes TJ and AJ. Counteracts VEGF-induced endothelial permeability by inhibiting phosphorylation of VE-cadherin. Up-regulates occludin and ZO-1 expression and prevents occludin phosphorylation, favoring occludin interaction with ZO-1. Suppresses VEGF-induced expression of ICAM-1 and VCAM-1.	[33,34,35,36,37,38,39]
Retinoic acid	Interacts with RAR in ECs, interferes with ShH and Wnt pathways. Increases expression of ZO-1, occludin, claudin-5, and VE-cadherin. Activates nuclear factor erythroid 2-related factor 2 (NRF2) pathway, which results in ICAM-1 and VCAM-1 decreased expression in BMECs.	[43,44,45,46,47,48,81]
Wnt Growth Factors	WNT/β-catenin pathway activation and nuclear translocation of β-catenin increase endothelial expression of claudin-3, claudin-5, and occludin reducing BBB permeability. Decrease caveolin-1 expression and decreased transcellular vesicular traffic in brain ECs.	[49,50,86]
Glial-derived Neurotrophic Factor	Binds on GDNF family receptor α1 and RET receptor kinase, activates sirtuin 1/ eNOS, PI3K/Akt, cAMP/PKA pathway and inactivates p38 MAPK, preventing TJ protein degradation; increases claudin-5, occludin and ZO-1 expression.	[52,53,54]
Fibroblast Growth Factor	Binds to FGFR1 and activates S1PR1, ERK, and PI3K/AKT/Rac-1 pathways preventing TJ and AJ proteins degradation. Increases astrocytic proliferation, influences astrocyte morphology.	[55,56,57,58,59,60,61]
Apolipoprotein E3	APOE4 binds on LRP1 in pericytes and induces MMP-9 secretion from pericyte via CypA/NFκB pathway. ApoE3 counteracts BBB disruption through MMP-9 activation by ApoE4.	[62,63,64]
Factors promoting BBB permeability		
Vascular Endothelial Growth Factor (VEGF)	Activation of nuclear factor κB (NF-κB) by TLRs and RAGE or activation of JAK2/STAT3/ HIF1α pathway are involved in VEGF production in astrocytes. Activation of HIF1α/VEGF/VEGFR2/ERK pathway is involved in the TNF-α-induced down-regulation of TJ proteins. IL-1β induces HIF1α-mediated VEGF expression. VEGF signaling increases BBB permeability through PI3K/Akt/eNOS, PLCγ/ PKC/ ERK, p38, and Src pathways, resulting in TJ-related protein down-regulation (occludin and claudin-5) and eNOS up-regulation. VEGF induces expressions of ICAM-1, VCAM-1, and MMP-9. eNOS activation and NO produce TJ and AJ complex disruption. Activation of Src and focal adhesion kinase result in phosphorylation and internalization of the VE-Cadherin and AJ breakdown.	[40,65,66,67,68,69,70]
Nitric oxide (NO) Reactive oxygen species (ROS)	NO production in reactive astrocytes after iNOS up-regulation is followed by activation of GMP/PKG and endothelial TJ protein down-regulation. ROS lead to arachidonic acids-mediated MMP activation and increased cytokine production. ROS induce down-regulation and degradation of TJ-related proteins through activation of the ROCK /MLC /MLCK pathway.	[61,80,86,87,88,89]
Matrix metalloproteinases (MMPs)	MMPs degrade the extracellular matrix proteins (collagen, fibronectin, and laminin), and TJ-related proteins. Astrocytic MMP-2 and MMP-9 stimulate NF-κB activation, leading to chemokine expression.	[61,72,73,74,75,76,77,90]
Glutamate	Decreased glutamate reuptake in reactive astrocytes results in excessive extracellular glutamate and increased MMP-2 and MMP-9 expression.	[71,77,78,86]
Endothelins (ETs) and other vasoactive mediators	Endothelin-1 (ET-1), arachidonic acids, and arachidonic acids metabolite PGE2 are up-regulated in reactive astrocyte via PLA2 activation. PGE2 modulates BBB integrity and induces endothelial migration via cAMP/PKA pathway activation. Increased expression of ET-1 in reactive astrocytes impairs BBB integrity through endothelial MMP-2, -9, and VEGF up-regulation. ET-1 triggers astrocytic AQP4 down-regulation, and affects ECs contact with astrocytic end-feet. ET-1 binding on endothelial ET-A and ET-B results in NF-κB activation, vascular inflammation, increased PGE2 production via COX2 activation, and immune cell migration due to up-regulated ICAM-1, and VCAM-1, and E-selectin.	[79,80,81,82,83,84]

## Abbreviations

ACE-1	Angiotensin converting enzyme-1
AHR	Aryl hydrocarbon receptor
AJ	Adherens junction
AKT (PKB)	Protein kinase B
AMPA	α-amino-3-hydroxy-5-methyl-4-isoxazolepropionic acid
ANG-1/2	Angiopoetin-1/2
ApoE	Apolipoprotein E
ATAC-seq	Transposase accessible chromatin sequencing
BAFF	B cell activating factor of the TNF family
BBB	Blood–brain barrier
BCR	B-cell receptor
BDNF	Brain-derived neurotrophic factor
BM	Basement membrane
CCL	Chemokine ligand
CD	Cluster of differentiation
CNS	Central nervous system
DhH	Desert Hedgehog
DNA	Deoxyribonucleic acid
ECs	Endothelial cells
ECM	Extracellular matrix
eNOS	Endothelial nitric oxide synthase
ER	Endoplasmic reticulum
ERK	Extracellular signal-regulated kinase
ET	Endothelin
ETR	Endothelin receptor
FGF	Fibroblast Growth Factor
GDNF	Glial-derived Neurotrophic Factor
cGMP	Cyclic guanosine monophosphate
GTP	Guanosine-5′-triphosphate
HAT	Histone acetyltransferase
HDAC	Histone deacetylases
HDM	Histone demethylase
HMT	Histone methyltransferase
ICAM-1	Intercellular adhesion molecule -1
IFN	Interferon
IGF-1	Insulin-Like Growth Factor
IKK	IkappaB kinase
IL	Interleukin
IRAK	Interleukin-1 receptor-associated kinase
IRF-1	Interferon regulatory factor-1
JAK	Janus kinase
HIF	Hypoxia-inducible factor
HMGB1	High mobility group box 1 protein
LaST	Large-area spatial transcriptomic
LIF	Leukemia inhibitory factor
LPS	Lipopolysaccharide
LRP1	Low density lipoprotein receptor-related protein 1
MAP	Mitogen-activated protein
MLC	Myosin light chain
MLCK	Myosin light chain kinase
MMP	Matrix metalloproteinase
mRNA	Messenger ribonucleic acid
miRNA	Micro ribonucleic acid
MRP	miRNAs after myeloid-related protein
NFAT	Nuclear factor of activated T-cells
NF-kB	Nuclear Factor kappa-light-chain-enhancer of activated B cells
NMDA	N-methyl-D-aspartate
NO	Nitric oxide
NVU	Neurovascular unit
PAK	Src–Rac1–p21-activated kinase
PHD2	Prolyl hydroxylase domain protein 2
PI3K	Phosphoinositide3 kinase
PK	Protein kinase
PL	Phospholipase
PTPN-2	protein tyrosine phosphatase non-receptor type 2
RA	Retinoic acid
RAGE	Advanced glycation end products
RNAs	Ribonucleic acids
ROS	Reactive oxygen species
ROCK	Rho-associated protein kinase
RRBS	Reduced representation bisulfite sequencing
scRNA-seq	Single cell RNA sequencing
snRNA-seq	Single nucleus RNA sequencing
STAT3	Signal transducer and activator of transcription 3
Smo	Signal transducer Smoothened
smFISH	Single-molecule fluorescent in situ hybridization
SOCS	Suppressor of cytokine signaling 1
SHh	Sonic Hedgehog
TAK	TGFβ-activated kinase 1
TCR	T-cell receptor
TGF-β	Transforming Growth Factor-β
TJ	Tight junction
TLR	Toll-like receptor
TNF	Tumor Necrosis Factor
VCAM-1	Vascular cell adhesion molecule
VE	Vascular endothelial
VEGF	Vascular Endothelial Growth Factor
ZO	Zonula occludens
